# Secondary Hemophagocytic Lymphohistiocytosis in a Rocky Mountain Spotted Fever Patient

**DOI:** 10.7759/cureus.105330

**Published:** 2026-03-16

**Authors:** César A Rojas Landa, Diego O Rojas Landa, Benjamin A Rojas Landa, Miriam N Martínez Brownell, Hanniel E Román Robles

**Affiliations:** 1 Internal Medicine, Hospital General de Mexicali, Mexicali, MEX; 2 Diagnostic and Therapeutic Imaging, Hospital General de Mexicali, Mexicali, MEX; 3 Emergency, Instituto Mexicano del Seguro Social, Mexicali, MEX; 4 General Medicine, Universidad Autónoma de Baja California, Mexicali, MEX; 5 General Medicine, Universidad Autónoma de Baja California, Valle de las Palmas, Tijuana, MEX

**Keywords:** fever, hemophagocytic, lymphohistiocytosis, rickettsia, rocky mountain spotted fever, spotted fever rickettsiosis

## Abstract

Hemophagocytic lymphohistiocytosis (HLH) is a rare and potentially fatal hyperinflammatory syndrome caused by uncontrolled activation of cytotoxic T lymphocytes, natural killer (NK) cells, and macrophages, leading to cytokine storm and multiorgan dysfunction. Due to its nonspecific clinical presentation and high mortality, early recognition and multidisciplinary management are essential.

We report the case of a 28-year-old woman admitted with fever, myalgias, arthralgias, and generalized exanthema. Initial laboratory evaluation revealed pancytopenia. Polymerase chain reaction (PCR) testing was positive for *Rickettsia*, and bone marrow examination demonstrated findings consistent with HLH. Despite appropriate management, the patient developed septic shock and died due to multiple complications, including multiorgan failure.

## Introduction

Hemophagocytic lymphohistiocytosis (HLH) is a potentially fatal and often underrecognized hyperinflammatory syndrome that can occur in both children and adults [1]. It is characterized by uncontrolled immune activation that may rapidly progress to multiorgan failure and death if not identified and treated promptly. Early recognition is essential due to its aggressive course and high mortality [1,2].

In HLH, an exaggerated inflammatory response driven by cytotoxic T lymphocytes and macrophage overactivation leads to excessive cytokine release. Natural killer (NK) cell dysfunction plays a central role in disease pathogenesis and contributes to characteristic laboratory abnormalities, including hyperferritinemia [3].

Primary HLH occurs predominantly in children and young adults and is associated with genetic defects affecting cytotoxic function, whereas secondary HLH is more commonly diagnosed in adults and typically develops in the context of infections, malignancies, autoimmune diseases, or immunosuppression [4,5].

In adults, HLH remains particularly challenging to diagnose because of its heterogeneous clinical presentation and significant overlap with sepsis and other severe inflammatory syndrome. Large cohort studies and registry-based analyses have shown that HLH is frequently underdiagnosed in adult populations, with delayed recognition associated with worse outcomes and increased mortality [4,6]. Recognition of key clinical and laboratory features, including persistent fever, cytopenias, splenomegaly, hyperferritinemia, and hypofibrinogenemia, is essential, though none of these findings are disease-specific.

The most widely used diagnostic framework is based on the HLH-2004 criteria, originally developed for pediatric populations and later extrapolated to adults, where their diagnostic performance is suboptimal [7]. Diagnosis requires either the identification of a pathogenic genetic mutation or fulfillment of at least five of eight clinical and laboratory criteria, including fever, splenomegaly, cytopenias, hypertriglyceridemia and/or hypofibrinogenemia, hemophagocytosis, decreased or absent NK cell activity, elevated ferritin levels, and increased soluble CD25 [7].

Despite the availability of these criteria, differentiation of HLH from related hyperinflammatory conditions - such as macrophage activation syndrome associated with rheumatologic diseases or severe sepsis - remains challenging due to overlapping clinical and laboratory features [8].

## Case presentation

A 28-year-old woman with a nine-year history of epilepsy on magnesium valproate and a two-year history of smoking (one cigarette/day) presented with a one-week history of fever, myalgias, arthralgias, asthenia, and adynamia. She later developed low back pain and received outpatient antibiotic treatment. One day before admission, she experienced clinical deterioration with fever up to 39.5°C and was brought to the hospital. Physical examination revealed generalized petechiae.

Initial laboratory evaluation revealed pancytopenia, abnormal liver function tests, and electrolyte disturbances, including hyponatremia and hypocalcemia. Triglyceride levels were elevated, and the peripheral blood smear demonstrated morphological abnormalities of erythrocytes. Ferritin levels were markedly increased. Additional findings included a positive direct Coombs test, and abdominal ultrasound revealed hepatosplenomegaly. Results of laboratory tests are summarized in Table 1.

Given the high clinical suspicion of rickettsial infection, empirical doxycycline therapy was initiated on the first day of hospitalization.

During hospitalization, the patient developed rapid hemodynamic deterioration requiring vasopressor support, along with neurological decline attributed to septic shock, which necessitated endotracheal intubation and transfer to the ICU. After fulfilling the diagnostic criteria for HLH, dexamethasone therapy was initiated in the ICU.

**Table 1:** Key laboratory findings

Test/Study	Result	Reference Range
Hemoglobin	7.77 g/dL	13.5–17.5 g/dL
Platelets	15.3×10³/µL	150–400×10³/µL
White Blood Cells	3.21×10³/µL	4.5–10×10³/µL
Total Bilirubin	2.9 mg/dL	0.20–1.30 mg/dL
Direct Bilirubin	1.9 mg/dL	0.0–0.40 mg/dL
Aspartate Aminotransferase	2,073 U/L	17–59 U/L
Alanine Aminotransferase	380 U/L	0.0–35 U/L
Gamma-Glutamyl Transferase	26 U/L	15–73 U/L
Lactate Dehydrogenase	2,617 U/L	125–618 U/L
Sodium	126 mmol/L	137–145 mmol/L
Calcium	6.2 mg/dL	8.4–10.2 mg/dL
Triglycerides	361 mg/dL	0–150 mg/dL
Ferritin	>3,000 ng/mL	4.0–104.20 ng/mL
Direct Antiglobulin Test	Positive	–
Hypochromia	Present	–
Elliptocytes	Present	–
Stomatocytes	Present	–
Echinocytes	Present	–

**Figure 1:**
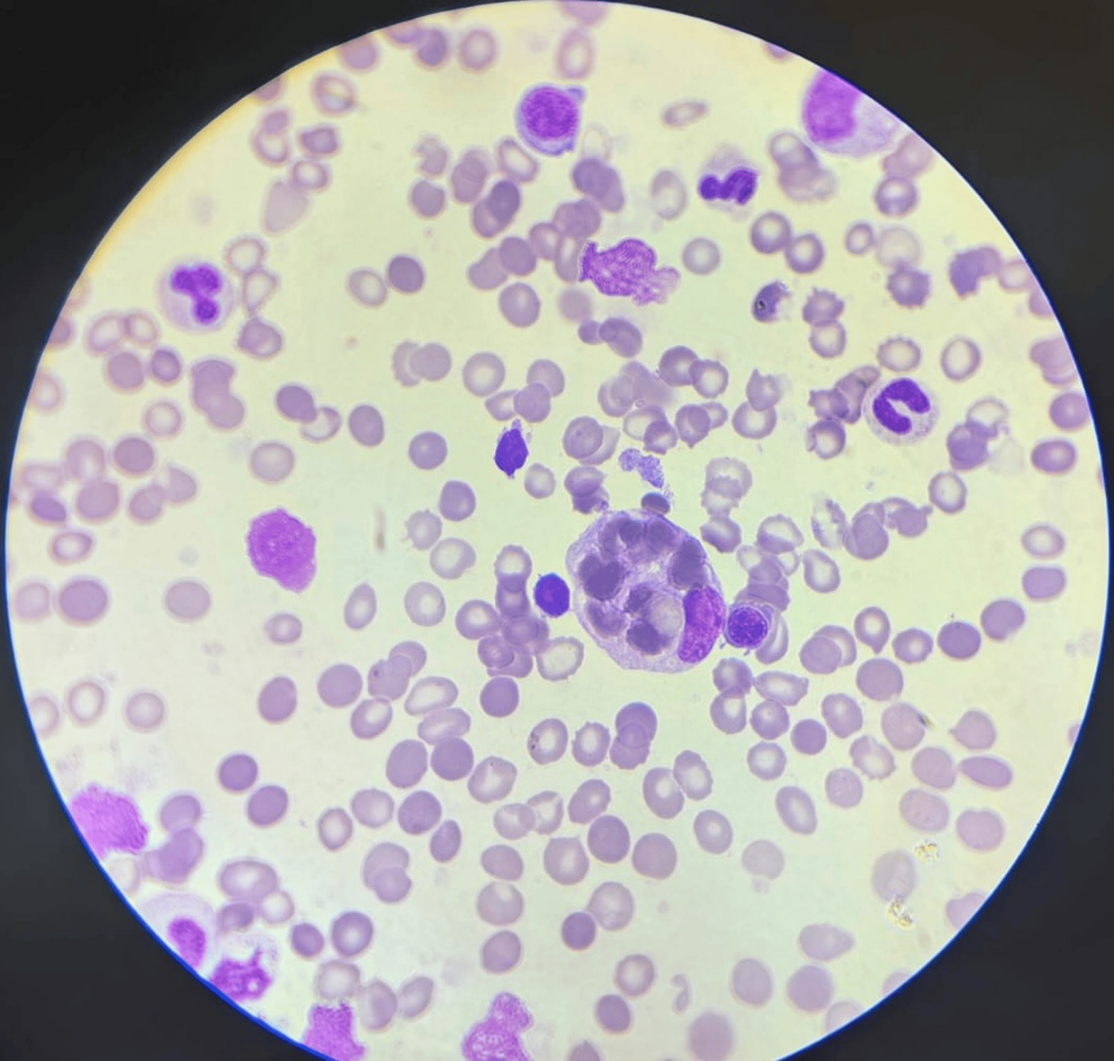
Peripheral blood smear stained with Wright–Giemsa and examined at high magnification. The background shows numerous erythrocytes, some with mild anisocytosis and preserved central pallor. Scattered mature leukocytes are present. Notably, a large cell compatible with an activated histiocyte is identified, showing abundant, pale, vacuolated cytoplasm containing formed blood elements, a finding suggestive of hemophagocytic activity.

**Figure 2:**
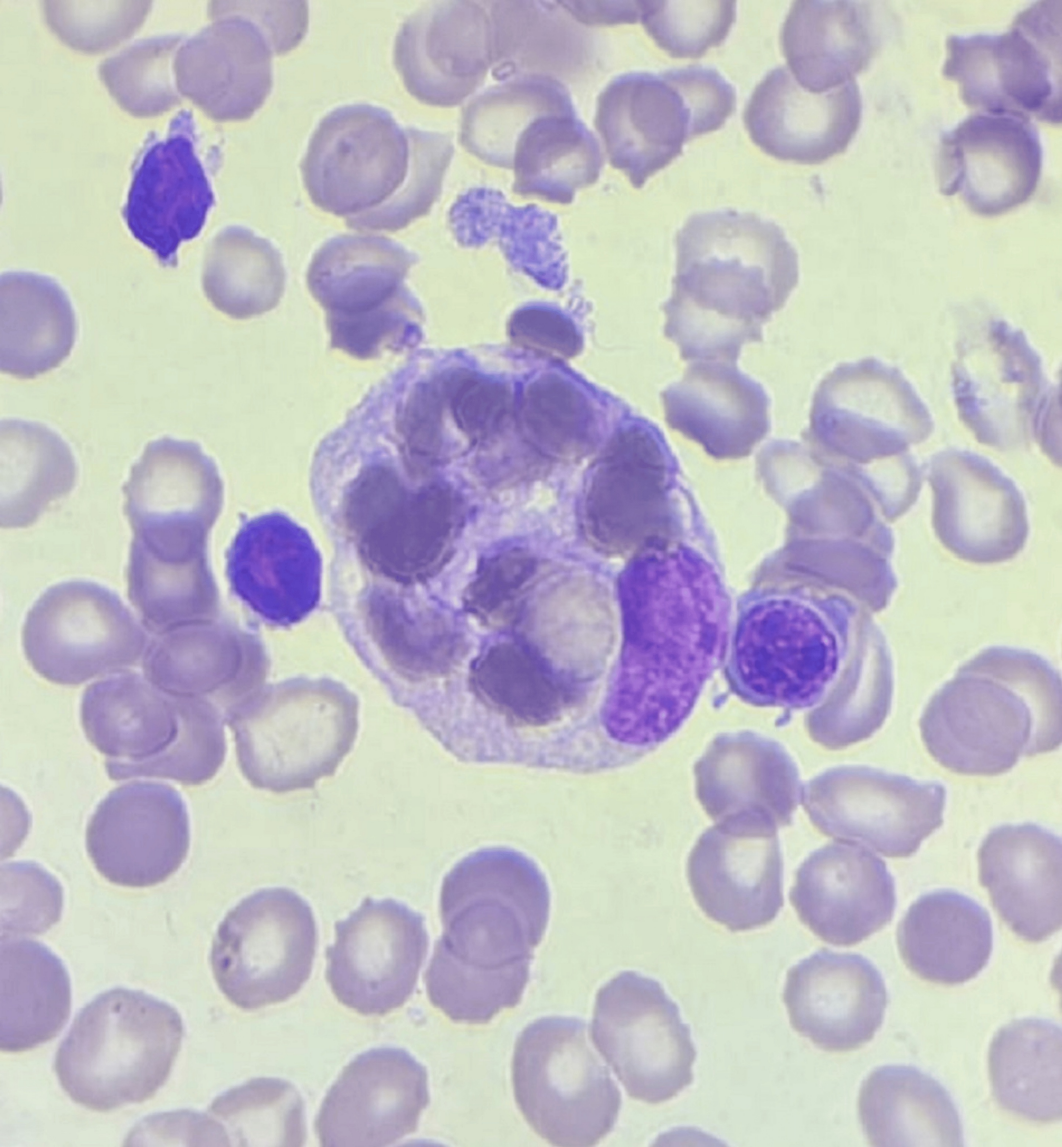
Digitally magnified view of the same peripheral blood smear, enabling detailed evaluation of histiocyte morphology. A large cell with abundant vacuolated cytoplasm is observed containing engulfed erythrocytes and cellular debris. The nucleus appears irregular and eccentrically located. This morphological finding is characteristic of hemophagocytosis and reflects the uncontrolled macrophage activation that defines HLH. HLH: Hemophagocytic lymphohistiocytosis

Hematology consultation was obtained, and bone marrow aspiration revealed hemophagocytosis (Figures 1, 2).

A diagnosis of HLH was established based on the HLH-2004 criteria. The patient fulfilled five diagnostic criteria, including persistent fever (>38.5°C), splenomegaly, cytopenias affecting three cell lineages, hyperferritinemia, and hypertriglyceridemia. Fibrinogen levels were not available at our institution; however, the diagnosis was established based on the fulfillment of six of the eight HLH-2004 criteria [7]. The HScore was not formally calculated due to the absence of some required laboratory parameters. Soluble CD25 and NK cell activity testing were unavailable.

Polymerase chain reaction (PCR) returned positive for *Rickettsia*, confirming Rocky Mountain spotted fever (RMSF). Despite treatment for RMSF, septic shock, and HLH, the patient developed multiorgan failure and died from complications. 

## Discussion

Diagnosing HLH remains a major clinical challenge due to its significant overlap with sepsis and other severe inflammatory syndromes. As a result, the condition is frequently underdiagnosed, particularly in adult patients [9]. Recognition of key clinical and laboratory features such as persistent fever, cytopenias, splenomegaly, hyperferritinemia, and hypofibrinogenemia is essential, although none of these findings are disease-specific. Early clinical suspicion and timely diagnosis are critical to initiating appropriate therapy and improving outcomes.

Several studies have demonstrated that adult patients often fail to meet classical diagnostic thresholds early in the disease course, contributing to delayed recognition and treatment [9,10]. Among laboratory abnormalities, hyperferritinemia, although nonspecific, has emerged as one of the most sensitive markers of HLH and is associated with disease severity and mortality, particularly when markedly elevated [6,11].

The HLH-2004 criteria remain the most widely used diagnostic framework; however, their application in adults is limited by reduced specificity and sensitivity [7,10]. Consequently, diagnosis should rely on an integrated assessment of clinical presentation, laboratory findings, and disease trajectory rather than strict adherence to diagnostic thresholds alone [7].

In adult patients, alternative diagnostic tools such as the HScore have been developed to improve diagnostic accuracy. The HScore integrates several clinical and laboratory variables to estimate the probability of reactive HLH and has shown good diagnostic performance in critically ill patients [10].

Hemophagocytosis on bone marrow examination has limited sensitivity and specificity and may be absent in early stages of the disease. Its absence does not exclude HLH and should not delay treatment initiation when clinical suspicion is high [8,12]. Bone marrow findings must therefore be interpreted in conjunction with clinical, laboratory, and immunological data.

Differentiation from related hyperinflammatory conditions, such as macrophage activation syndrome associated with rheumatologic diseases or systemic inflammatory response syndrome, remains challenging, as these entities share overlapping clinical and laboratory features [8].

Severe infections are recognized triggers of secondary HLH, and rickettsial infections have been increasingly reported as potential precipitants of macrophage activation. The intense immune response induced by *Rickettsia rickettsii*, including endothelial infection and intense systemic cytokine release, may lead to dysregulated macrophage activation and hemophagocytosis. Although severe sepsis may present with overlapping hyperinflammatory features, the presence of persistent cytopenias affecting multiple cell lines, marked hyperferritinemia, hypertriglyceridemia, splenomegaly, and histopathologic evidence of hemophagocytosis supported the diagnosis of HLH in this patient rather than sepsis alone [3,6,7].

Treatment strategies focus on rapid control of the hyperinflammatory response, management of the underlying trigger, and prevention of organ failure. While traditional approaches have relied on immunosuppressive and cytotoxic therapies, emerging evidence supports the use of targeted immunomodulatory agents, including cytokine-directed therapies and Janus kinase inhibitors, in selected cases, particularly in refractory disease or when conventional therapies are contraindicated [3,5,6]. Early initiation of therapy remains the most important determinant of survival.

## Conclusions

HLH is a severe and potentially fatal hyperinflammatory syndrome whose diagnosis remains a significant clinical challenge, particularly in adults, due to its nonspecific presentation and substantial overlap with sepsis and other hyperinflammatory conditions. This case highlights the importance of maintaining a high index of suspicion in patients presenting with persistent fever, cytopenias, organ dysfunction, and markedly elevated inflammatory markers, especially hyperferritinemia.

Diagnostic evaluation in adults requires an integrated interpretation of clinical, laboratory, and immunological findings. The absence of hemophagocytosis on initial bone marrow evaluation does not exclude the diagnosis and should not delay the initiation of treatment.

Early recognition and timely intervention, aimed both at controlling the hyperinflammatory response and addressing the underlying trigger, are critical to improving outcomes. This case underscores the need for a multidisciplinary approach and reinforces the importance of considering HLH in the differential diagnosis of adults with unexplained systemic inflammatory response and multiorgan dysfunction.
